# Effects of exercise programmes delivered using video technology on physical performance and falls in people aged 60 years and over living in the community: a systematic review and meta-analysis

**DOI:** 10.1136/bmjopen-2024-092775

**Published:** 2025-04-30

**Authors:** Fadhia Adliah, Abigail J Hall, Victoria Goodwin, Sarah Lamb

**Affiliations:** 1 Public Health and Sport Sciences, University of Exeter Faculty of Health and Life Sciences, Exeter, UK; 2 Faculty of Nursing, Physiotherapy, Hasanuddin University, Makassar, Indonesia

**Keywords:** Digital Technology, Exercise, Aged

## Abstract

**Abstract:**

**Objectives:**

This systematic review and meta-analysis synthesised the evidence and evaluated the effect of exercise programmes delivered using instructional videos compared with control on physical performance and falls in community-dwelling older people aged 60 years and older.

**Design:**

A systematic review and meta-analysis conducted following the Preferred Reporting Items for Systematic Reviews and Meta-Analyses guidelines.

**Data sources:**

MEDLINE, EMBASE, CINAHL, PsycINFO, The Cochrane Central Register of Controlled Trials, TRIP and PEDro. Grey literature sources included theses and dissertations from Ethos and ProQuest.

**Eligibility criteria:**

Studies were included if they involved community-dwelling older people (aged >60 years) participating in exercise programmes delivered through instructional videos.

**Data extraction and synthesis:**

Treatment effects were estimated using a random-effects model, reporting 95% CIs, mean differences (MD) and standardised MDs (SMD, Hedges’ g) for outcomes measured in different units. The risk of bias was assessed using ROB2, and the certainty of evidence was evaluated using the Grading of Recommendations Assessment, Development and Evaluation (GRADE) approach.

**Results:**

A total of 7487 records were screened, with 16 studies (n=1910) meeting the inclusion criteria. Meta-analysis of 11 studies revealed significant effects of video-delivered exercise programmes in lower extremity strength (SMD=0.35, 95% CI 0.11 to 0.59; I^2^=70.35%, p<0.001, GRADE moderate quality), balance (SMD=0.45, 95% CI 0.07 to 0.83; I^2^=85.07%, p=0.02, GRADE low quality), mobility (MD=0.96, 95% CI 0.46 to 1.46; I^2^=53.31%, p<0.001, GRADE moderate quality) and physical performance SMD=0.36, 95% CI 0.17 to 0.56; I^2^=13.49%, p<0.001, GRADE moderate quality). No evidence of an effect of video-delivered exercise programmes on fear of falling was found (SMD=0.5, 95% CI −0.30 to 1.29; I^2^=95.48%, p=0.22, GRADE very low quality). There were insufficient data for reporting falls.

**Conclusions:**

Video-delivered exercise programmes improved physical performance, particularly lower extremity strength, balance and mobility, with low to moderate quality evidence. There is uncertainty about the effect of video-delivered exercise programmes on the number of falls, number of fallers and fear of falling.

**PROSPERO registration number:**

CRD42023415530.

**Strengths and limitations of this study**

- This is the first systematic review and meta-analysis specifically examining the role of video demonstrations in supporting exercise programmes.
- Conducted according to the Preferred Reporting Items for Systematic Reviews and Meta-Analyses guidelines and a prespecified International Prospective Register of Systematic Reviews-registered protocol. The methodological quality of the included reviews was assessed using standardised measures.
- The findings provide valuable insights for future digital or remote-based interventions.
- Potential limitations include the availability and heterogeneity of the existing evidence and variations in methodological quality.

## Introduction

 Regular exercise and physical activity offer numerous benefits for older adults, including the prevention and management of age-related conditions, improved mobility, enhanced mental well-being and a better quality of life.^1 2^ In particular, exercise has been shown to effectively reduce falls, even in vulnerable older populations, either alone or in combination with other interventions.^3^

To minimise fall risk, older adults should engage in tailored exercise programmes that incorporate multicomponent exercises, including muscle strengthening and balance training.^3 4^ These programmes should progressively increase in challenge and be performed regularly, including muscle-strengthening activities at least twice a week, along with functional balance training supplemented by brisk walking at least 3 days per week.^3^,^5^

Despite the availability of best-practice clinical guidelines for exercise-based fall prevention, participation and adherence among older adults remain low.^6^ Barriers include personal factors such as lack of motivation, boredom, fear of injury and pre-existing health conditions.^7^,^9^ Additionally, environmental and logistical challenges such as poor access to exercise facilities, limited transportation options, safety concerns, weather conditions and cost further hinder participation.^10^,^13^

To address these barriers, technology has been increasingly integrated into physical activity programmes to enhance engagement and adherence, particularly in rehabilitation settings.^14 15^ Over time, technology-based exercise interventions have expanded to community and residential settings, offering accessible and affordable ways to promote physical activity in older adults.^16^ Advances in digital technology, including computers, tablets and smartphones, now provide convenient and flexible options for delivering exercise programmes.^16^,^18^

Among these technological advancements, video-based exercise demonstrations have gained popularity as an option to guide older adults through exercise routines. Compared with text-based instructions, videos provide clear visual demonstrations, verbal instructions and often motivating background music, which can enhance comprehension, engagement and adherence.^19^,^22^ The accessibility of video-based exercises has further improved with the widespread availability of the internet and smartphones, allowing for remote participation at a relatively low cost. This became particularly relevant during the COVID-19 pandemic when video-based interventions enabled the continuation of exercise programmes despite social restrictions.^23 24^

A recent systematic review and meta-analysis by Lee *et al*^25^ examined the effects of fall prevention interventions using information and communication technology (ICT), including telehealth, computerised balance training, exergaming, mobile applications, virtual reality and cognitive-behavioural training. Their findings demonstrated that ICT-based interventions, particularly telehealth and exergames, improved balance, reduced fall risk and enhanced physical function in older adults. However, the review encompassed a broad range of ICT-based interventions and did not specifically evaluate the effectiveness of video-based exercise demonstrations.

Given the increasing adoption of video technology for exercise training, there remains a need to systematically review and synthesise the evidence on its effectiveness in improving physical performance and reducing falls in older adults. Therefore, this systematic review and meta-analysis aims to address this gap by focusing specifically on the role of video demonstrations in supporting exercise programmes. The objective is to evaluate the effectiveness of video-based exercises compared with usual care or non-exercise intervention in enhancing physical performance and reducing falls.

## Method

This systematic review and meta-analysis has been reported according to the Preferred Reporting Items for Systematic Reviews and Meta-Analyses (PRISMA) guidelines.^26^ The PRISMA checklist can be found in onlinesupplemental materials 1  2. The protocol was registered with the International Prospective Register of Systematic Reviews (PROSPERO) under registration number CRD42023415530. Patients and/or the public were not involved in the design, or conduct, or reporting, or dissemination plans of this research.

### Eligibility criteria

We included all randomised controlled trials (RCTs) published in English to ensure accurate data extraction and interpretation while minimising the risk of translation errors. Eligibility criteria were defined using the PICO (Population, Intervention, Comparator, Outcomes) framework,^26^ and studies had to meet the following criteria for inclusion.

#### Population

Community-dwelling older adults (male and/or female) aged 60 years or older. Studies were eligible if the sample’s mean age was at least 60 years. Studies focusing on hospitalised or institutionalised older adults, as well as those exclusively involving individuals with specific diseases or conditions (eg, Parkinson’s disease, stroke), were excluded.

#### Intervention

Exercise programmes using prerecorded instructional videos (online or offline) to demonstrate exercises. Studies using synchronous instructional videos, such as live streaming, video calls or video conferencing, were excluded. Video-based exercise programmes could be supplemented with home visits or in-person interactions with practitioners.

#### Comparator

No exercise intervention or a non-exercise control intervention, such as receiving leaflets, links to physical activity promotion websites or physical activity guidelines.

#### Outcomes

The primary outcome was physical performance, defined as the observed ability to perform tasks related to transfer and mobility (eg, sit-to-stand, walking). Other related terms included physical function, functional ability or functional performance. Secondary outcomes included fall-related variables such as the number of falls, number of fallers and fear of falling.

### Data source

We systematically searched MEDLINE, EMBASE, CINAHL, PsycINFO, the Cochrane Central Register of Controlled Trials, TRIP and PEDro for articles published between 2000 and 2025. The initial search on 17 May 2023 covered studies from 2000 to 2023, and an update on 17 March 2025 using the same search strategy included recent studies from 2023 to 2025.

The search also included grey literature to identify unpublished research material, specifically theses and dissertations accessed through Ethos and ProQuest. Additionally, reference lists of included studies were manually searched for further eligible studies, and citation tracking (both backward and forward) was conducted using Google Scholar.

The search strategy employed Boolean operators (AND, OR, NOT), filters (date range) and other relevant limits. A full, detailed search strategy, including precise search terms, Boolean logic, filters and limits applied for each database and register, as well as the date of each database search, is provided in online supplemental material 3.

### Study selection

**Figure 1:**
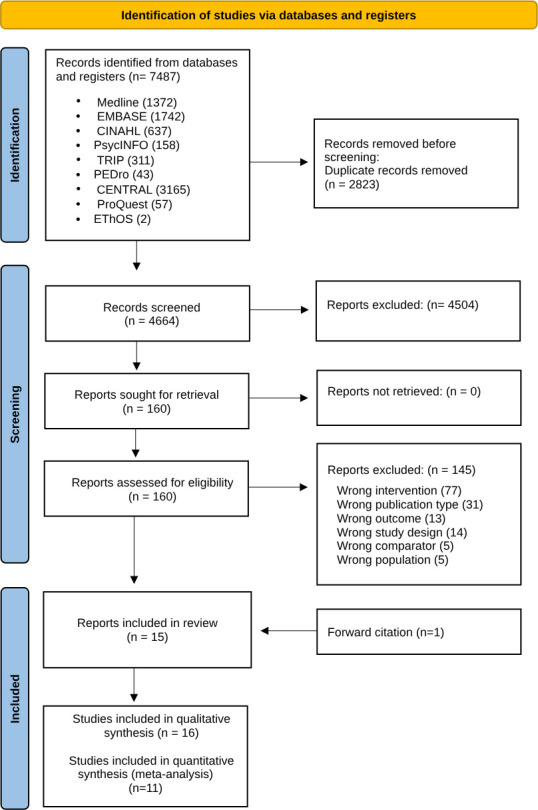
PRISMA flow diagram. PRISMA, Preferred Reporting Items for Systematic Reviews and Meta-Analyses.

All retrieved papers were first deduplicated using EndNote V.20 and then exported to RAYYAN^27^ for manual screening. Two reviewers (FA and AJH) independently conducted the screening process. Initially, titles and abstracts from the selected databases were screened, followed by full-text screening based on the predetermined inclusion and exclusion criteria. A third reviewer (VG) resolved any disagreements. Studies that did not meet the eligibility criteria were excluded, with reasons for exclusion documented in the PRISMA flow diagram^26^ (figure 1).

### Data extraction

Data were extracted using an electronic data extraction form. Study authors were contacted via email for additional information on missing data. One reviewer (FA) extracted the data, and another (AJH) independently verified it. Extracted data included the following: author, year of publication, country, participant characteristics (sample size, age, sex, health status), study design, recruitment sources, eligibility criteria, setting, exercise type and components, dose, mode of delivery, video characteristics (technology used, method of delivery) and adherence. Primary and secondary outcome data were collected for preintervention and postintervention time points. If multiple follow-up time points were reported, the earliest was selected. Any disagreements between the two reviewers were resolved through discussion with a third author (VG).

### Methodological quality assessment

We used the Cochrane Risk of Bias tool (RoB2) to assess bias in each included study, categorising it as low risk, high risk or some concerns.^28^ To evaluate overall evidence quality, we applied the Grading of Recommendations Assessment, Development and Evaluation (GRADE) system via the GRADEPro website, following the Cochrane Handbook for Systematic Reviews.^29 30^ GRADE rates evidence as high, moderate, low or very low based on the risk of bias, imprecision, indirectness and inconsistency. Quality was downgraded if (1) most data came from high-risk studies, (2) outcomes had fewer than 400 participants, (3) evidence did not directly address PICO or (4) heterogeneity was high (I² >50%). The first author conducted the risk of bias and quality assessment, which was then reviewed by the team. Discrepancies were resolved through discussion until a consensus was reached.

### Data analysis

Meta-analysis was conducted using STATA V.18.0 software with random-effects models. This approach was chosen due to the heterogeneity of the population, measurements and interventions. Meta-analysis was performed only when at least three studies were available for comparison per outcome.

For continuous variables measured in the same units, treatment effects were calculated using mean differences (MDs). When different measurement units were used, standardised MDs (SMDs, Hedges’ g) were applied.^31^ Effect estimates were derived from post-score means and SDs (or their estimates), with 95% CIs for between-group change scores. Because some measurements indicated that higher scores reflected better physical performance, while others indicated the opposite, scores from studies where higher values represented worse physical performance were multiplied by −1 to ensure consistency before inclusion in the meta-analysis.^31^

Heterogeneity among studies was visually assessed using forest plots and quantified with the I² statistic. An I² value of 50% or lower indicated homogeneity, while values above 50% suggested substantial heterogeneity. Statistical significance was set at p<0.05. SMDs were calculated by dividing the difference in means between groups by the pooled SD at the postintervention time point. SMD values of 0.20, 0.50 and 0.80 were interpreted as small, medium and large effects, respectively.^32^ Publication bias was assessed visually using funnel plots. When insufficient data were available for meta-analysis, a narrative synthesis was conducted.

### Patient and public involvement

Patients and/or the public were not involved in the design, or conduct, or reporting, or dissemination plans of this research.

## Results

### Search outcome

The initial search was conducted on 17 May 2023 and updated on 17 March 2025. A total of 7487 records were identified through database and manual searches. After removing 2823 duplicates and excluding 4504 records based on title and abstract screening, 160 reports underwent full-text review. Of these, 15 studies met the inclusion criteria, and an additional study was identified through forward citation tracking, bringing the total to 16 studies. The PRISMA flow diagram (figure 1) illustrates this process.

### Study characteristics

16 studies published between 2007 and 2025 in English were included. These studies were conducted in the UK,^33^ Australia,^22 34^ Spain,^19^ Greece,^20^ France,^35^ Italy,^36^ Denmark,^37^ Japan,^32 38^ Thailand,^21^ Taiwan^39^ and China.^40^ Additionally, one study from the USA generated three reports.^41^,^43^ Table 1 provides a summary of the study characteristics, with further details available in online supplemental material 4.

**Table 1:** Summary of the characteristics of included studies

Study	Sample (n); % female; mean age	Exercise components	Duration; session length; frequency	Setting; exercise mode	Video exercise; media; device
Vestergaard *et al*^37^	53; 100%; 81±3.3	Muscle strength, balance, flexibility and endurance	5 months; 26 min; 3×week	Home; individual; unsupervised	Entirely video; offline; video player
Haines *et al*^34^	50; 60.4%; 80.9±6.5	Muscle strength, balance	2 months; 13 min	Home; individual; supervised	Entirely video; offline; Digital Video Disc (DVD) player
Yamada *et al*^32^	84; 80.5%; 83±6.7	Muscle strength, balance, agility and dual tasks	6 months; 20 min; 2×week	Centre; group; supervised	Entirely video; offline; DVD player
McAuley *et al*^41^	260; 71.52%; 70.62±0.4	Muscle strength, balance and flexibility	6 months; 3×week	Home; individual; unsupervised	Entirely video; offline; DVD player
Wójcicki *et al*^43^	237; 71.5%; 70.6±0.4	Muscle strength, balance and flexibility	12 months follow-up; 3×week	Home; individual; unsupervised	Entirely video; offline; DVD player
Benavent-Caballer *et al*^19^	51; 69%; 69.1±4	Lower extremity strengthening, balance, mobility, flexibility, endurance	4 months; 45 min; 3×week	Centre; group; supervised	Entirely video; offline; DVD player
Boongird *et al*^21^	417; 86.6%; 74.08	Lower extremity strengthening, stretching and balance training	6 months; 60 min; 2–3×week	Home; individual; supervised	Entirely video; offline; Video Disk Recorder (VDR)
Roberts *et al*^42^	153; 73.6%; 70±4.98	Muscle strength, balance and flexibility	24 months follow-up; 3×week	Home; individual; unsupervised	Entirely video; offline; DVD player
Liang *et al*^33^	30; 67%; 71.1±3.6	Functional tasks, muscle strength, balance, tai chi	1 month; 2×day	Home; individual; unsupervised	Entirely video; online; smartphone/tablet and website platform
Mézière *et al*^35^	35; 83.3%; 90	Muscle strength, balance, functional tasks, joint mobilisation exercises	3 months; 2×week	Home; individual; supervised	Partially video (combined with face-to-face exercise); offline; tablet
Lytras *et al*^20^	150; 90.7%; 70	Lower extremity strengthening, balance, flexibility	6 months; 45 min; 5×week	Centre and home; both group and individual; supervised	Partially video (combined with face-to-face exercise); offline; Television or computer
Fyfe *et al*^22^	19; 67%; 69.8±3	Lower extremity strengthening, balance, functional tasks	1 month; 9 min; 3×day	Home; individual; unsupervised	Entirely video; online; smartphone/tablet and website platform
Chang *et al*^39^	167; 70.1%; 67.6±7.86	Resistance, static balance, dynamic balance, speed-walking	4 months; 60 min; 2–3×week	Centre and home; both group and individual; supervised	Partially video (combined with face-to-face exercise); online; smartphone and LINE chat application
Suzuki *et al*^38^	15; 33.3%	Slow squats, one-legged stance	3 months; 15 min; daily	Home; individual; supervised	Entirely video; online; smartphone and YouTube application
Ferrari *et al*^36^	73; 49%; 66.89±5.93	Muscle strength and balance	6 months; 30 min; 3×week	Home; individual; supervised	Entirely video; online; tablet and website platform
Zhou *et al*^40^	116; 25%; 84.4±3.2	Muscle strength and balance	12 months; 30 min; 3×week	Home; individual; supervised	Entirely video; online; smartphone and WeChat application

### Participants

The included studies had sample sizes ranging from 15 to 417 participants, with a total of 1910. The mean age of participants ranged from 67 to 90 years, and all were older adults living in the community. One study included only female participants,^37^ while 15 studies included both males and females, though two had fewer female participants.^38 40^

### Risk of bias

**Figure 2:**
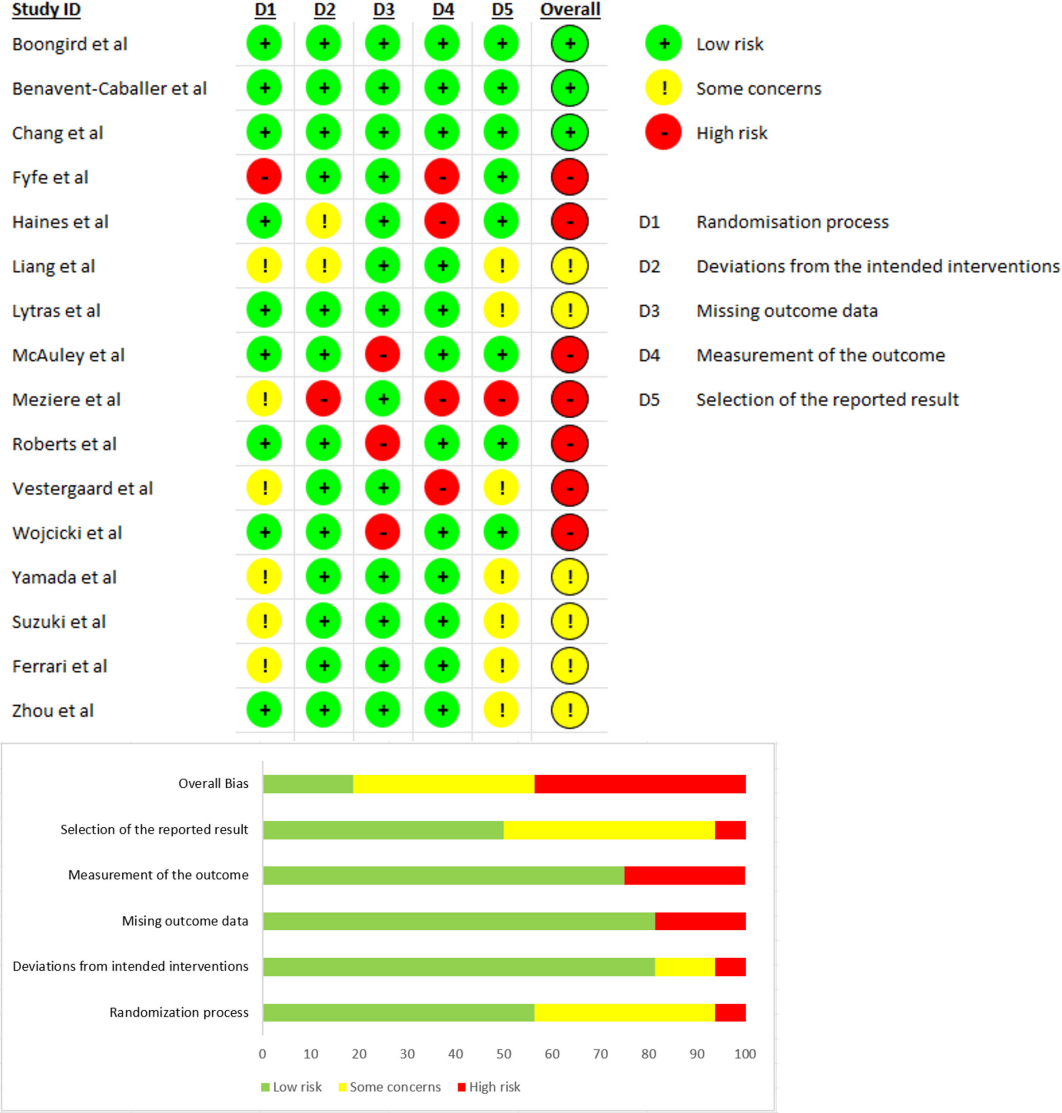
Risk of bias of included studies.

Three studies had a low risk of bias, six had some concerns and seven had a high risk of bias. Further details are shown in figure 2.

### Intervention

All included studies implemented multicomponent exercises incorporating both strength and balance. Five studies added flexibility exercises to the training component,^19^,^2137 39 41^ three studies added functional tasks exercise,^22 33 35^ three added endurance exercise^19 37 39^ and one added joint mobilisation exercises.^35^

The duration of intervention follow-up ranged from 1 month to 2 years. Four studies reported multiple follow-up time points: Haines *et al* and Lytras *et al* measure outcomes at two time points (2 and 6 months and 3 and 6 months, respectively),^20 34^ while Boongird *et al* and McAuley *et al* had three follow-up assessments (3, 6 and 12 months and 6, 12 and 24 months, respectively).^2141^,^43^

Exercise programme doses were varied, with a frequency of two to three times per week being the most commonly prescribed,^1932 35^,^37 39^ and 20–45 min being the most commonly used duration.^1920 32 35^,^37 40^ Some studies reported progressive training loads by increasing difficulty levels and using ankle cuff weights.^19^,^2234 35 41^ Monitoring strategies included telephone calls, exercise diaries and face-to-face visits. Three studies implemented the Otago Exercise Programmes, which required at least four home visits.^19^,^21^

Two delivery methods were used for prerecorded exercise videos. 10 studies (62.5%) provided offline access through digital video discs (DVDs) or videotapes,^19^,^2132 34 35 37 41^ while the remaining 6 (37.5%) used online delivery via smartphones, apps or websites.^2233 36 38^,^40^ More recent studies (since 2020) favoured online methods: Chang *et al*,^39^ Zhou *et al*^40^ used smartphone-based messaging apps to send exercise videos. Fyfe *et al*^22^ and Ferrari *et al*^36^ provided videos and guidelines via a website platform.

Eight studies (50%) compared video-delivered exercise to non-exercise interventions, such as providing educational materials on healthy lifestyles, fall prevention resources or a home helper without an exercise programme.^2021 33 35 39 41^,^43^ The remaining studies (50%) compared with no intervention at all.^1922 32 34 36^,^38 40^

### Outcome measures

Several studies assessed physical performance using a single measurement, such as the Short Physical Performance Battery^19 39 41^ or Physical Performance Test.^37^ However, some studies evaluated individual components of physical performance, including strength, balance and mobility.

Lower extremity strength was assessed using the Five Times Sit-to-Stand test^19 21 22 32 33 37 39 40^ and the 30 s Chair Stand Test.^20^ Balance was measured using the Berg Balance Scale,^19^,^21^ one leg stand,^33 39^ semi tandem stand^37^ and the Balance Outcome Measure for Elder Rehabilitation.^34^ Functional mobility was assessed using the Timed Up and Go (TUG) test.^19^,^2132 39^ Fear of falling was assessed using the Fall Efficacy Scale International (FES-I),^21^ Short FES-I^20^ or Activities-specific Balance Confidence Scale.^34^

### Effects of video-delivered exercise programmes on physical performance

**Figure 3:**
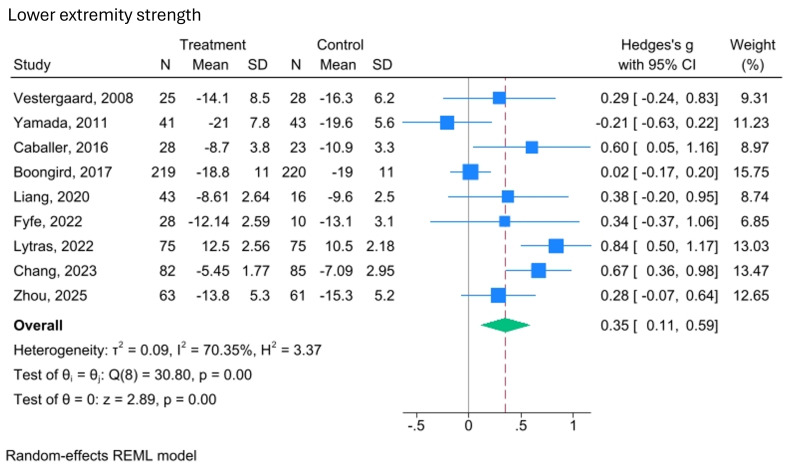
Forest plot of the effect of video-delivered exercise programmes on lower extremity strength in older adults. Meta-analysis of nine studies shows Hedges’ g effect sizes with 95% CIs. Blue squares represent individual effects (weighted by size), and the diamond indicates the pooled effect (Hedges’ g=0.35; 95% CI 0.11 to 0.59). Heterogeneity is notable (I²=70.35%, τ²=0.09) and the overall effect is significant (z=2.89, p<0.001). A random-effects model was used, with variance estimated by restricted maximum likelihood (REML).

Physical performance, as we define it, is a broad category encompassing strength, balance and mobility. While not all included studies use the same definition, they assess components that align with ours. The effects of video-delivered exercise programmes on these aspects of physical performance are illustrated in figures3^6^.

**Figure 4:**
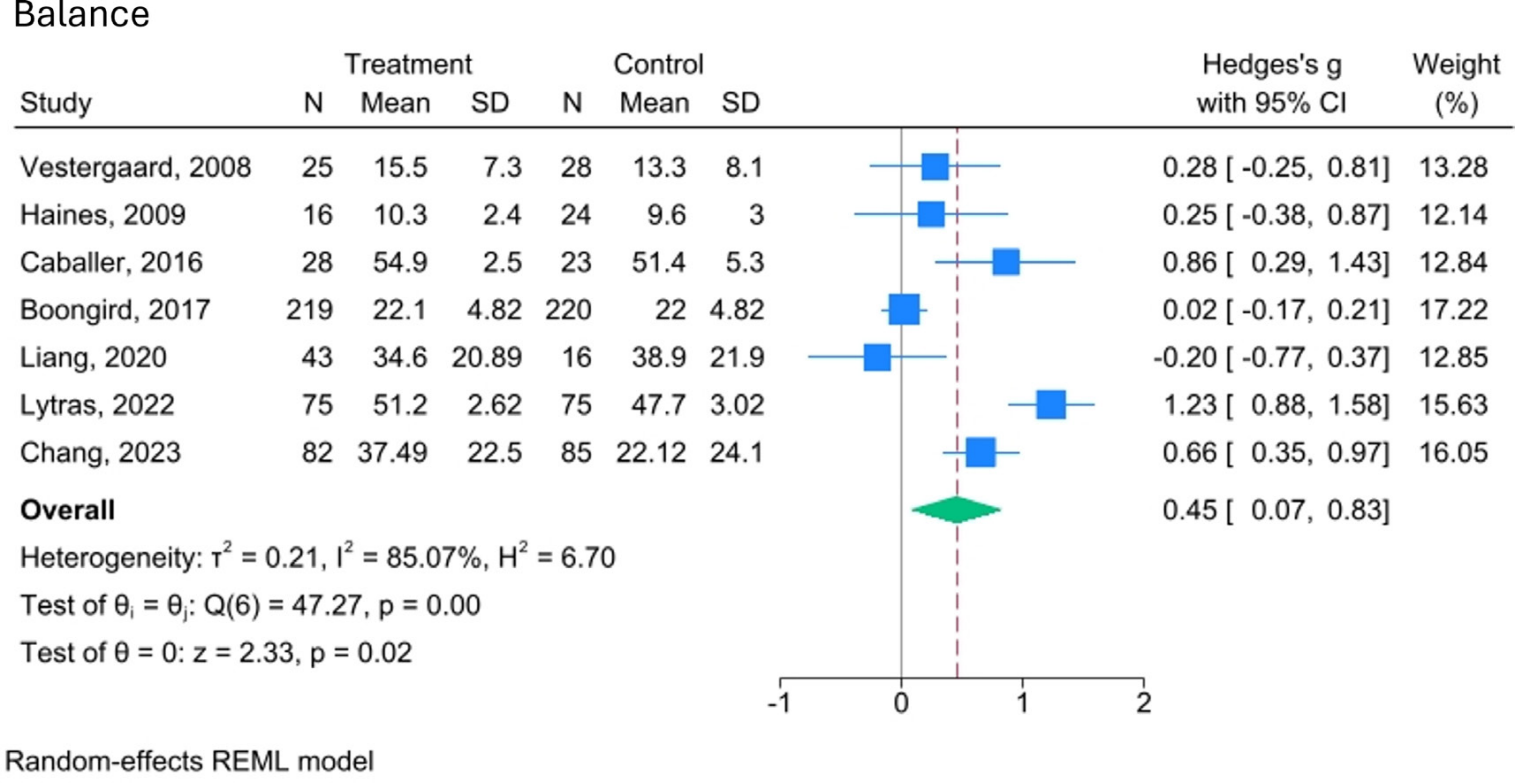
Forest plot of the effect of video-delivered exercise programmes on balance in older adults. Meta-analysis of seven studies shows Hedges’ g effect sizes with 95% CIs. Blue squares indicate individual effects (weighted by size), and the diamond represents the pooled effect (Hedges’ g=0.45; 95% CI 0.07 to 0.83). Heterogeneity is high (I²=85.07%, τ²=0.21), and the overall effect is significant (z=2.33, p=0.02). A random-effects model was used, with variance estimated by restricted maximum likelihood (REML).

**Figure 5:**
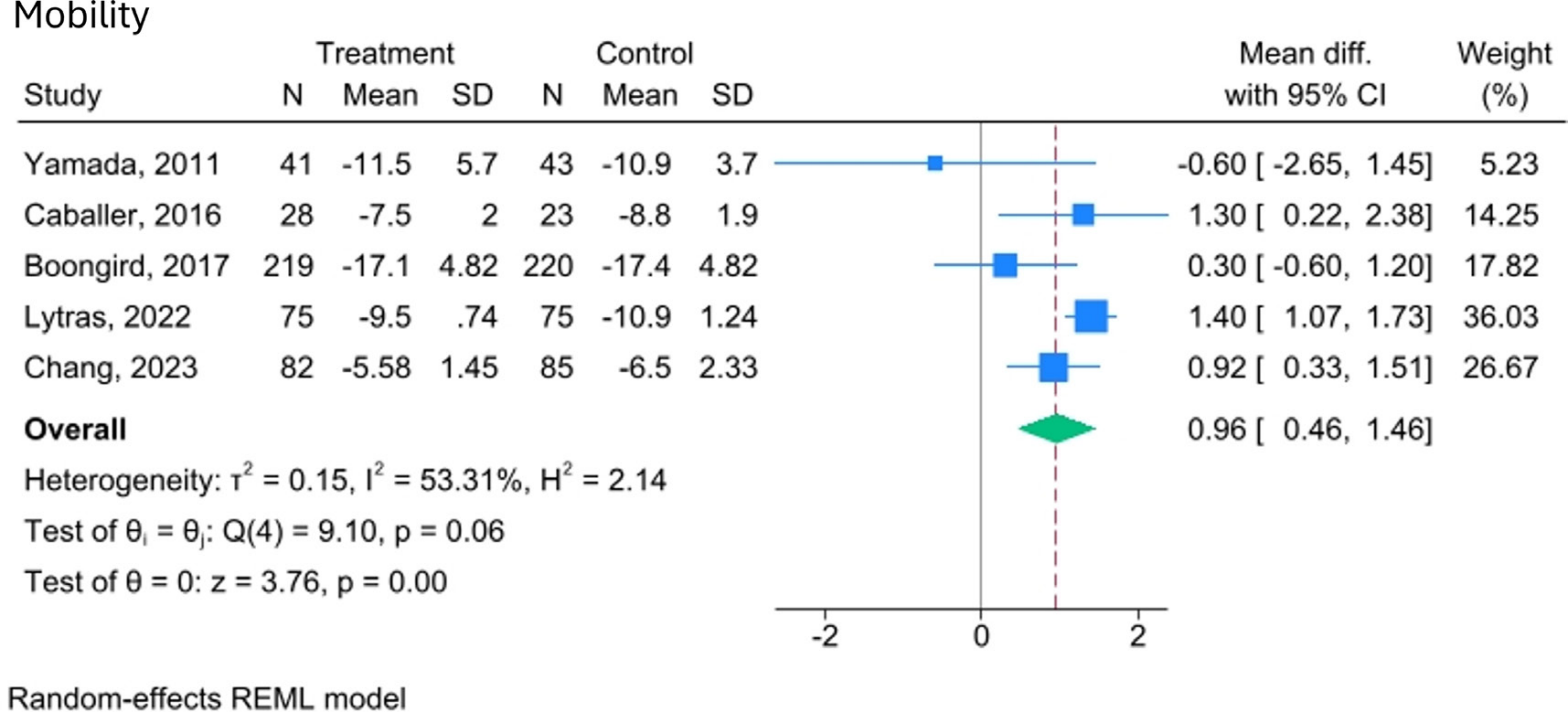
Forest plot of the effect of video-delivered exercise programmes on mobility in older adults. Meta-analysis of five studies shows mean difference effect sizes with 95% CIs. Blue squares represent individual effects (weighted by size), and the diamond indicates the pooled effect (mean difference=0.96; 95% CI 0.46 to 1.46). Heterogeneity is moderate (I²=53.31%, τ²=0.15), and the overall effect is significant (z=3.76, p=0.00). A random-effects model was used, with variance estimated by restricted maximum likelihood (REML).

**Figure 6:**
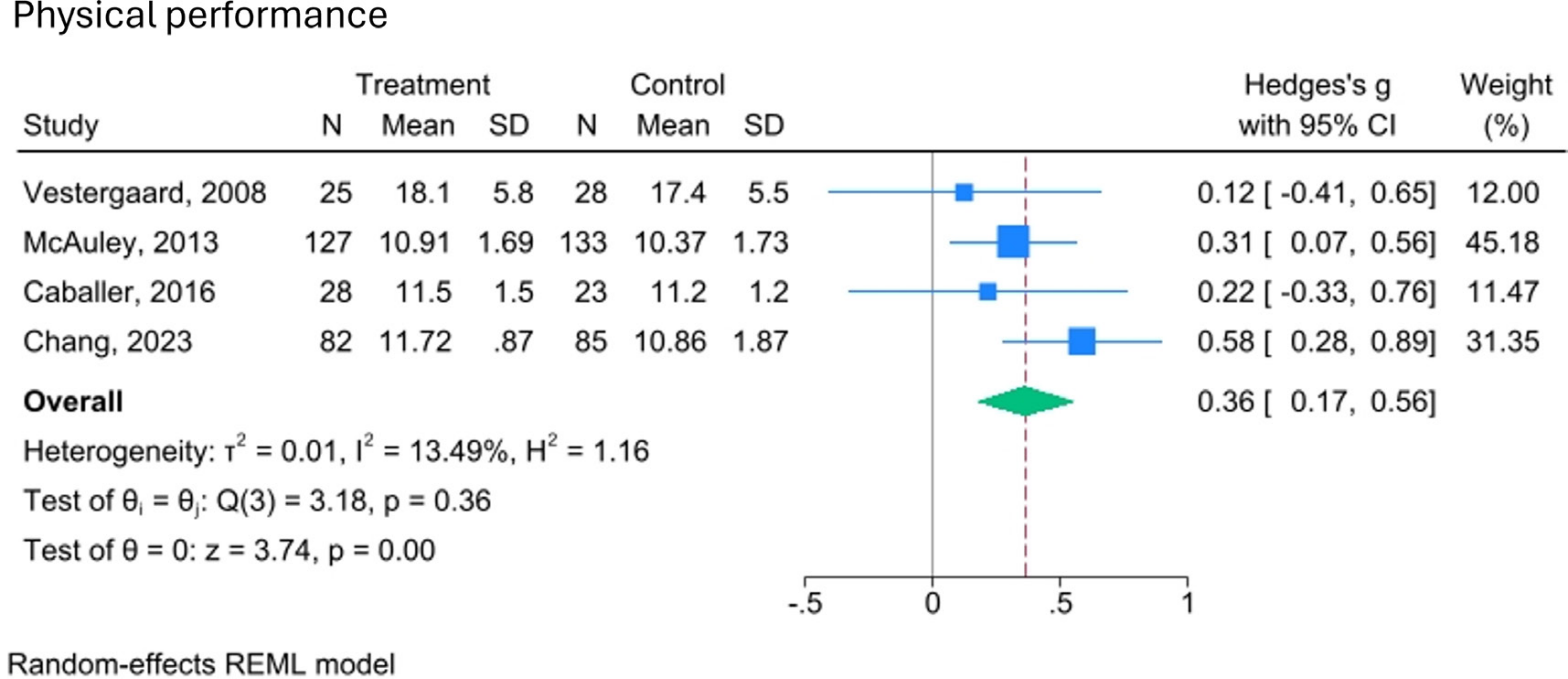
Forest plot of the effect of video-delivered exercise programmes on physical performance in older adults. Meta-analysis of four studies shows Hedges’ g effect sizes with 95% CIs. Blue squares represent individual effects (weighted by size), and the diamond indicates the pooled effect (Hedges’ g=0.36; 95% CI 0.17 to 0.56). Heterogeneity is low (I²=13.49%, τ²=0.01), and the overall effect is significant (z=3.74, p=0.00). A random-effects model was used, with variance estimated by restricted maximum likelihood (REML).

The pooled analysis of nine trials (n=1165) assessing lower extremity strength indicates a small but statistically significant improvement compared with the control group (SMD=0.35, 95% CI 0.11 to 0.59; I²=70.35%, p<0.001). This finding is supported by moderate-quality evidence (GRADE).

For balance, a pooled analysis of seven trials (n=959) shows a small to moderate, statistically significant effect in favour of video-delivered exercise programmes compared with control (SMD=0.45, 95% CI 0.07 to 0.83; I²=85.07%, p=0.02). However, this result is supported by low-quality evidence (GRADE).

The meta-analysis of five trials (n=891) evaluating the effects of video-delivered exercise on mobility found a statistically significant improvement compared with the control group (MD=0.96, 95% CI 0.46 to 1.46; I²=53.31%, p<0.001), supported by moderate-quality evidence (GRADE).

Additionally, four trials (n=531) assessing overall physical performance reported a small but statistically significant effect in favour of video-delivered exercise (SMD=0.36, 95% CI 0.17 to 0.56; I²=13.49%, p<0.001). These pooled results are also supported by moderate-quality evidence (GRADE).

A summary of the quality of evidence is provided in table 2.

**Table 2:** Summary of the quality of evidence

Outcome	Number of trials	Risk of bias*	Inconsistency†	Imprecision‡	Effect size (95% CI), I^2^, p value	Certainty
Lower extremity strength	9	–	–	–	SMD=0.35 (0.11 to 0.59); I^2^=70.35%, <0.001	⨁⨁⨁Moderate§
Balance	7	–	–	–	SMD=0.45 (0.07 to 0.83), I^2^=85.07%, 0.02	⨁⨁Low¶
Physical performance	4	**–**	–	–	SMD=0.36 (0.17 to 0.56), I^2^=13.49%, <0.001	⨁⨁⨁Moderate**
Mobility	6	–	–	–	MD=0.96 (0.46 to 1.46), I^2^=53.31%, <0.001	⨁⨁⨁Moderate§
Fear of falling	3	–	–	–	SMD=0.5 (−0.30 to 1.29), I^2^=95.48%, 0.22	⨁Very low††

* wWe downgraded if >25% of included trials had a high risk of bias.

† wWe downgraded if there was statistical heterogeneity or wide confidence intervalCI.

‡ wWe downgraded if there were <400 participants.

§ Reason for downgrade: statistical heterogeneity.

¶ Reason for downgrade: statistical heterogeneity, >25% of included trials had a high risk of bias.

** Reason for downgrade: >25% of included trials had a high risk of bias.

†† Reason for downgrade: statistical heterogeneity, wide CI, >25% of included trials had a high risk of bias.

MD, mean difference; SMD, standardised MD.

### Effects of video-delivered exercise programmes on fall-related variables

Four studies reported fear of falling.^20 21 34^ ^40^ Haines *et al*^34^ and Zhou *et al*^40^ reported that there was no difference in fear of falling scores of video-delivered exercise intervention vs control. Meanwhile, Boongird *et al*^21^ and Lytras *et al*^20^ found a statistically significant effect after 6 months of video-delivered exercise intervention compared with control. However, when the meta-analysis was performed, the pooled effect still indicated that there was no difference in fear of falling between video-delivered exercise programmes and control (SMD=0.5, 95% CI −0.3 to 1.29; I^2^=95.48%, p=0.22, n=760), with very low-quality evidence.

This review included only three studies that reported on fall rate and number of fallers, which was insufficient for a meta-analysis. All three studies observed a reduction in falls and the number of fallers in their intervention groups compared with controls. Boongird *et al*^21^ and Zhou *et al*^40^ reported fewer falls in the intervention group over a 1-year follow-up, while Haines *et al*^34^ observed a similar trend over 6 months. However, these differences did not reach statistical significance.

### Adverse events

Adverse events were reported in six studies (37.5%).^21 22 33 34 40 41^ No major adverse events were associated with the intervention. Reported minor adverse events included muscle pain, muscle discomfort and knee joint pain.

### Adherence to the video-delivered exercise programmes

11 studies (68.7%) reported adherence to exercise programmes using various indicators to evaluate adherence. The included studies defined adherence as follows: (1) the proportion of completed exercise sessions per person; (2) the proportion of participants who attended the exercise session; (3) the average number of exercise days per week; (4) the percentage of participants exercising for more than 120 min per week or (5) the total of video playbacks. Boongird *et al* reported relatively low adherence, with only 29.6% of participants exercising for more than 120 min per week in the first (3 month) follow-up.^21^ In contrast, Liang *et al* recorded the highest adherence, with 90% of participants completing the prescribed exercise intervention over 4 weeks.^33^ However, overall adherence to video-delivered exercise programmes remains uncertain due to heterogeneity in measurement methods and interpretation.

## Discussion

This systematic review evaluated the effects of video-delivered exercises on physical performance and falls in community-dwelling older adults. The quality of evidence varied across outcomes. While several studies had a strong randomisation process, some raised concerns regarding sequence generation and allocation concealment. Additionally, one study^22^ showed a significant difference in baseline characteristics, with the control group having more comorbidities. However, the absence of a p value for these differences led to a high risk of bias assessment.

Considering the heterogeneity of the population, measurements and interventions, the random effects model was chosen. Four meta-analyses showed differences in physical performance outcomes between participants who received video-delivered exercises. Although the measurement methods varied, process measures were favourable for video-delivered exercises, with a small effect size observed for physical performance with low to moderate-quality evidence. However, in some important outcomes including the number of falls, number of fallers and fear of falling, the quality of literature was poor, and fewer studies reported those outcomes, making it difficult to draw robust conclusions.

Despite the uncertainty surrounding fall-related outcomes, our findings indicate that video-delivered exercises positively influence lower extremity strength, balance, mobility and overall physical performance. Strength-focused exercises included chair sit-to-stand, calf raises, hip abductor strength exercises and resistance-based hip and knee movements, as seen in the Otago exercise programme. Balance exercises frequently demonstrated in the video included one-leg stands, clock stepping, marching on the spot, tandem walking and multidirectional walking (side and backwards).

While these improvements reached statistical significance, their clinical relevance remains uncertain. The observed effect sizes were small, particularly for balance (SMD=0.45, 95% CI 0.07 to 0.83). Lower extremity strength improvements (SMD=0.35, 95% CI 0.11 to 0.59) were supported by moderate-quality evidence, providing greater confidence in this result. In contrast, balance outcomes were supported by low-quality evidence, reducing the certainty of their impact. Improvements in mobility (MD=0.96 s, 95% CI 0.46 to 1.46) were statistically significant and supported by moderate-quality evidence, yet they fell below the minimal clinically important difference threshold of 2.1 s established in previous research.^44^ This suggests that while improvements were measurable, they may not translate into meaningful functional benefits for older adults. The discrepancy may stem from differences in participant health status (eg, healthier vs frailer individuals) and the measurement tools used. Although the TUG test is widely used for fall risk assessment, it may not be sensitive enough to detect changes in highly functional older adults, resulting in limited observed effects.^45^

Overall physical performance also showed a small but statistically significant improvement (SMD=0.36, 95% CI 0.17 to 0.56), supported by moderate-quality evidence, indicating that video-delivered exercises can enhance multiple aspects of physical function. However, the small effect size suggests that while beneficial, the real-world impact may be limited. Future research should explore the clinical implications of these findings, particularly regarding their role in fall prevention.

With advances in technology, video-based interventions have emerged as a practical tool for delivering structured exercise programmes to older adults. Video instruction offers clear visual demonstrations and, in some cases, background music to enhance engagement.^19 37 46^ This review found that both online and offline delivery methods were viable. Offline methods require additional devices such as DVD players and television screens, though computers with internal video players can serve as an alternative. Online methods, by contrast, rely on internet-based platforms such as websites, video-sharing applications and smartphone chat apps for accessibility and distribution.

Adherence to video-delivered exercise programmes may be influenced by video quality and presentation style.^41 42^ High levels of satisfaction with video clarity and instruction have been linked to better adherence. Conversely, unclear instructions or poor-quality visuals may discourage engagement, leading some older adults to prefer face-to-face demonstrations.^33^ Additionally, videos featuring older adults as demonstrators may enhance relatability and motivation, thereby improving adherence rates.^35^

Despite some limitations, our findings suggest that video-delivered exercise programmes are a feasible, accessible and beneficial approach to improving physical performance in community-dwelling older adults. The convenience of remote exercise delivery may be particularly valuable for individuals with mobility limitations or those living in areas with limited access to in-person exercise programmes. The value of video-based exercise was further emphasised during the COVID-19 pandemic when restrictions on community-based activities highlighted the need for alternative, home-based solutions.

### Study limitations

While we believe this is the first systematic review and meta-analysis of RCT-based evidence on video-delivered exercise for community-dwelling older people, several limitations should be acknowledged. First, limiting the included articles to those published in English may have resulted in a smaller number of studies and the potential exclusion of relevant research published in other languages or studies that did not explicitly mention video-based exercise. Second, the number of studies reporting fall-related outcomes was insufficient, which may have affected the certainty of conclusions regarding falls and fear of falling. Third, substantial heterogeneity was observed due to variations in follow-up periods and measurement criteria. However, because of the small number of included studies, we were unable to conduct meaningful subgroup analyses, limiting our ability to explore potential variations in effect sizes.

## Conclusions

This review suggests that video-delivered exercise programmes can effectively improve physical performance, including lower extremity strength, balance and mobility, in community-dwelling older adults. These findings highlight the potential of video-based interventions as an alternative to traditional in-person exercise programmes, particularly for individuals with mobility limitations or those in remote areas. However, the impact of video-delivered exercise on falls and the fear of falling remains uncertain due to the limited number of studies reporting these outcomes. Given the positive trends in digital technology, future research should prioritise high-quality trials examining fall-related outcomes, long-term adherence and optimal delivery methods to maximise both engagement and clinical effectiveness.

## Supplementary material

10.1136/bmjopen-2024-092775 online supplemental file 1

10.1136/bmjopen-2024-092775 online supplemental file 2

10.1136/bmjopen-2024-092775 online supplemental file 3

10.1136/bmjopen-2024-092775 online supplemental file 4

## Data Availability

All data relevant to the study are included in the article or uploaded as supplementary information.
