# Supplementary material for: Effects of exercise programmes delivered using video technology on physical performance and falls in people aged 60 years and over living in the community: a systematic review and meta-analysis
Source: BMJ Open. 2025 Apr 30;15(4):e092775. doi: 10.1136/bmjopen-2024-092775 (PMC12049923; doi:10.1136/bmjopen-2024-092775)
Supplement: online supplemental file 1 [file bmjopen-15-4-s001.pdf]

Full search strategies

This supplementary file outlines our full list of search strategies used throughout the review across multiple databases and time points. These are outlined below.

PICOS	Criteria
Population<br>(older adults)	Participants are community-dwelling older adults (male or female) who are 60 years or older. Studies will be considered if the mean age is at least 60 years.<br><br>Exclusion criteria :<br>(1) Hospitalised or institutional-based older adults<br>(2) Study that only includes people with a specific disease or condition (e.g., Parkinson's disease, stroke)
Intervention<br>(video-supported home exercise)	The intervention is a home-based exercise programme delivered using pre-recorded instructional videos. The videos can be accessed online (e.g., websites, apps) or offline (e.g., CD, DVD, flash disc) and played at home using a television, CD/DVD player, computer, laptop, tablet, or mobile phone/smartphone. Exercise programmes may be supplemented with home visits or in-person interactions with practitioners.<br>Exclusion criteria:<br>(1) virtual reality video;<br>(2) video game-based exercise; or<br>(3) synchronous video-based exercise (e.g., videoconferencing, video call)
Comparator<br>(no intervention or non-exercise intervention)	There is at least one control group or comparator that:<br>(1) Receive no intervention. No intervention means participants were told to continue with their everyday routines and received no intervention at all; or<br>(2) Receive non-exercise or non-physical therapy interventions. This can be verbal or written educational intervention (e.g., a leaflet, books, a link to physical activity promotion web, or physical activity guidelines).<br><br>Exclusion criteria: The study without a control group will be excluded.
Outcomes<br>(physical performance, fear of falling, number of falls, number of fallers)	The primary outcome is physical performance (other terms may be physical function, functional ability, or functional performance)<br>Secondary outcomes are fall-related variables including fear of falling, number of falls, and number of people who fall.
Study design	All randomised controlled trials (RCTs) including randomised pilot and randomised feasibility-controlled trials, randomised crossover trials, and cluster randomised controlled trials.

List of searches

1. MEDLINE (via Ovid)
2. EMBASE (via Ovid)
3. CINAHL
4. PsycINFO (via OVID)
5. The Cochrane Central Register of Controlled Trials (Cochrane Library)
6. TRIP
7. PEDro
8. Ethos
9. ProQuest

1. **MEDLINE (via Ovid)**
   The search was conducted on 17 May 2023 and was updated on 17 March 2025.

   Search 1
   Date searched: 17 May 2023
   Records downloaded: 1066

1	aged/ or "aged, 80 and over"/ or frail elderly/	3,444,504
2	"older adult*".ab,ti.	114,285
3	"elder*".ab,ti.	298,577
4	"senior*".ab,ti.	50,564
5	"geriatric*".ab,ti.	57,438
6	older people.ab,ti.	36,506
7	older.ab,ti.	543,259
8	aged 65.ab,ti.	32,633
9	older person.ab,ti.	1,601
10	1 or 2 or 3 or 4 or 5 or 6 or 7 or 8 or 9	3,820,835
11	"exercise*".ab,ti.	343,939
12	"training*".ab,ti.	533,080
13	"home*".ab,ti.	612,932
14	physical activity.ab,ti.	140,586
15	"program*".ab,ti.	1,057,679
16	11 or 12 or 13 or 14 or 15	2,326,382
17	"video*".ab,ti.	163,929
18	taped.ab,ti.	3,515
19	"DVD*".ab,ti.	2,068
20	"website*".ab,ti.	38,399
21	web-based.ab,ti.	41,882
22	remote.ab,ti.	93,258
23	"tablet*".ab,ti.	62,039
24	"ipad*".ab,ti.	1,861
25	"smartphone*".ab,ti.	21,307
26	"phone*".ab,ti.	48,030
27	"youtube*".ab,ti.	3,602
28	"computer*".ab,ti.	336,450
29	"television*".ab,ti.	15,595
30	"application*".ab,ti.	1,558,716
31	ehealth.ab,ti.	4,108
32	mhealth.ab,ti.	5,340
33	17 or 18 or 19 or 20 or 21 or 22 or 23 or 24 or 25 or 26 or 27 or 28 or 29 or 30 or 31 or 32	2,264,013
34	"physical perform*".ab,ti.	13,033
35	"physical function*".ab,ti.	31,894
36	"functional perform*".ab,ti.	5,447
37	"functional abilit*".ab,ti.	8,024
38	balance.ab,ti.	266,181
39	strength.ab,ti.	366,969

40	"fall*".ab,ti.	243,741
41	"mobilit*".ab,ti.	176,655
42	"physical abilit*".ab,ti.	1,841
43	34 or 35 or 36 or 37 or 38 or 39 or 40 or 41 or 42	1,052,376
44	10 and 16 and 33 and 43	3,439
45	"random*".ab,ti.	1,408,604
46	44 and 45	1,090
47	limit 46 to yr="2000 -Current"	1,066

Updating the search

Before publication, the search was updated to reflect more recent literature, building upon the initial search conducted in 2023. The same strategy and keywords were used, but the publication date was restricted to articles published between 1 January 2023, and 12 March 2025, to capture any additional relevant studies.
Date searched: 17 March 2025
Additional records downloaded: 306

2. **EMBASE (via Ovid)**
The search was conducted on 17 May 2023 and was updated on 17 March 2025.

Search 1
Date searched: 17 May 2023
Records downloaded: 1373

1	aged/ or "aged, 80 and over"/ or frail elderly/	3,567,609
2	"older adult*".ab,ti.	146,669
3	"elder*".ab,ti.	426,746
4	"senior*".ab,ti.	72,230
5	"geriatric*".ab,ti.	91,835
6	older people.ab,ti.	45,369
7	older.ab,ti.	768,725
8	aged 65.ab,ti.	46,552
9	older person.ab,ti.	2,164
10	1 or 2 or 3 or 4 or 5 or 6 or 7 or 8 or 9	4,142,389
11	"exercise*".ab,ti.	466,485
12	"training*".ab,ti.	714,978
13	"home*".ab,ti.	831,106
14	physical activity.ab,ti.	191,545
15	"program*".ab,ti.	1,412,611
16	11 or 12 or 13 or 14 or 15	3,099,891
17	"video*".ab,ti.	236,696
18	taped.ab,ti.	5,069
19	"DVD*".ab,ti.	3,618
20	"website*".ab,ti.	57,567
21	web-based.ab,ti.	58,419
22	remote.ab,ti.	115,703
23	"tablet*".ab,ti.	104,620

24	"ipad*".ab,ti.	3,852
25	"smartphone*".ab,ti.	27,682
26	"phone*".ab,ti.	74,374
27	"youtube*".ab,ti.	4,784
28	"computer*".ab,ti.	417,759
29	"television*".ab,ti.	17,241
30	"application*".ab,ti.	1,786,674
31	ehealth.ab,ti.	4,704
32	mhealth.ab,ti.	5,640
33	17 or 18 or 19 or 20 or 21 or 22 or 23 or 24 or 25 or 26 or 27 or 28 or 29 or 30 or 31 or 32	2,739,416
34	"physical perform*".ab,ti.	17,789
35	"physical function*".ab,ti.	50,470
36	"functional perform*".ab,ti.	7,337
37	"functional abilit*".ab,ti.	11,817
38	balance.ab,ti.	340,192
39	strength.ab,ti.	425,307
40	"fall*".ab,ti.	318,485
41	"mobilit*".ab,ti.	207,407
42	"physical abilit*".ab,ti.	2,707
43	34 or 35 or 36 or 37 or 38 or 39 or 40 or 41 or 42	1,293,496
44	10 and 16 and 33 and 43	4,890
45	"random*".ab,ti.	1,954,348
46	44 and 45	1,396
47	limit 46 to yr="2000 -Current"	1,373

Search 2

Before publication, the search was updated to reflect more recent literature, building upon the initial search conducted in 2023. The same strategy and keywords were used, but the publication date was restricted to articles published between 1 January 2023, and 14 March 2025, to capture any additional relevant studies.
Date searched: 17 March 2025
Additional records downloaded: 369

3. **CINAHL**

The search was conducted on 17 May 2023 and was updated on 17 March 2025.
Date searched: 17 May 2023
Records downloaded: 552

Searching on abstract and title using these keywords:
Older adults OR older people OR older person OR (older women or aging women or elderly women) OR (older men or older males or elderly men) OR elderly OR senior OR geriatric OR aged 65 OR aged OR (frailty or frail elderly) AND<br>Exercise OR training OR home OR physical activity OR program* AND<br>Video* OR taped OR DVD* OR website* OR web-based OR remote OR tablet* OR computer* OR ipad* OR phone* OR television OR TV or smartphone* or youtube* or application or ehealth or mhealth or digital health AND

Physical perform* OR physical function OR functional perform* OR functional ability* OR balance OR strength OR fall OR mobilit* OR physical ability* AND Random*
Limiters - Published Date: 20000101-20231231<br>Expanders - Apply equivalent subjects<br>Search modes - Boolean/Phrase

Before publication, the search was updated to reflect more recent literature, building upon the initial search conducted in 2023. The same strategy and keywords were used, but the publication date was restricted to articles published between 1 January 2023, and 17 March 2025, to capture any additional relevant studies.
Date searched: 17 March 2025
Additional records downloaded: 85

4. **PsycINFO (via Ovid)**
The search was conducted on 17 May 2023 and was updated on 17 March 2025.

Search 1
Date searched: 17 May 2023
Records downloaded: 134

1	aged/ or "aged, 80 and over"/ or frail elderly/	1828
2	"older adult*".ab,ti.	60768
3	"elder*".ab,ti.	71222
4	"senior*".ab,ti.	30650
5	"geriatric*".ab,ti.	17345
6	older people.ab,ti.	16145
7	older.ab,ti.	179499
8	aged 65.ab,ti.	10047
9	older person.ab,ti.	1014
10	1 or 2 or 3 or 4 or 5 or 6 or 7 or 8 or 9	264256
11	"exercise*".ab,ti.	73941
12	"training*".ab,ti.	293168
13	"home*".ab,ti.	180821
14	physical activity.ab,ti.	40601
15	"program*".ab,ti.	443385
16	11 or 12 or 13 or 14 or 15	876444
17	"video*".ab,ti.	74306
18	taped.ab,ti.	3879
19	"DVD*".ab,ti.	1324
20	"website*".ab,ti.	15143
21	web-based.ab,ti.	15612
22	remote.ab,ti.	14398
23	"tablet*".ab,ti.	5896
24	"ipad*".ab,ti.	1316
25	"smartphone*".ab,ti.	6585
26	"phone*".ab,ti.	30920

27	"youtube*".ab,ti.	1659
28	"computer*".ab,ti.	97864
29	"television*".ab,ti.	16366
30	"application*".ab,ti.	189872
31	ehealth.ab,ti.	1013
32	mhealth.ab,ti.	1133
33	17 or 18 or 19 or 20 or 21 or 22 or 23 or 24 or 25 or 26 or 27 or 28 or 29 or 30 or 31 or 32	432307
34	"physical perform*".ab,ti.	1978
35	"physical function*".ab,ti.	7483
36	"functional perform*".ab,ti.	1118
37	"functional abilit*".ab,ti.	2761
38	balance.ab,ti.	49858
39	strength.ab,ti.	68472
40	"fall*".ab,ti.	55423
41	"mobilit*".ab,ti.	22426
42	"physical abilit*".ab,ti.	1041
43	34 or 35 or 36 or 37 or 38 or 39 or 40 or 41 or 42	199402
44	10 and 16 and 33 and 43	618
45	"random*".ab,ti.	236116
46	44 and 45	137
47	limit 46 to yr="2000 -Current"	134

Search 2

Before publication, the search was updated to reflect more recent literature, building upon the initial search conducted in 2023. The same strategy and keywords were used, but the publication date was restricted to articles published between January 2023, and 17 March 2025, to capture any additional relevant studies.

Date searched: 17 March 2025

Additional records downloaded: 24

5. **CENTRAL (Cochrane Library)**

The search was conducted on 17 May 2023 and was updated on 17 March 2025.

Search 1

Date searched: 17 May 2023

Records downloaded: 2170

#1	Older adult* OR older person OR older people OR elderly OR geriatric OR senior* OR aged 65	146512
#2	Exercise* OR training OR home OR physical activity OR program*	342321
#3	Video* OR taped OR DVD* OR website OR web-based OR remote OR tablet* OR ipad* OR smartphone* OR phone* OR youtube* OR computer* OR television* OR application* OR ehealth OR mhealth OR digital health	239200

#4	Balance OR strength OR fall* OR mobilit* OR physical perform* OR physical function* OR physical ability* OR functional perform* OR functional abilit*	186206
#5	Random*	1220919
#6	#1 AND #2 AND #3 AND #4 AND #5<br>With publication year from 2000 to 2023, in Trials	2170

Search 2
Before publication, the search was updated to reflect more recent literature, building upon the initial search conducted in 2023. The same strategy and keywords were used, but the publication date was restricted to articles published between January 2023, and December 2025, to capture any additional relevant studies.
Date searched: 17 March 2025
Additional records downloaded: 995

6. **PEDro**
The search was conducted on 17 May 2023 and was updated on 17 March 2025.

Date searched: 17 May 2023
Records downloaded: 40

**Search strategy:**
Title and abstract: Video-based exercise<br>Subdiscipline: Gerontology<br>Method: clinical trial<br>Published since: 2000

The search was updated on 17 March 2025 using the same strategy and keywords. The publication date was limited to articles published from 1 January 2023 to 3 March 2025.
Additional records downloaded: 3

7. **TRIP**
The search was conducted on 17 May 2023 and was updated on 17 March 2025.
Date searched: 17 May 2023
Records downloaded: 309

**Search strategy:**
(Older adult* OR older person OR older people OR elder OR geriatric OR senior* OR aged 65) AND (Exercise* OR training OR home OR physical activity OR program*) AND (Video* OR taped OR DVD* OR website OR web-based OR remote OR tablet* OR ipad* OR smartphone* OR phone* OR youtube* OR computer* OR television* OR application* OR ehealth OR mhealth OR digital health) AND (Random*)<br><br>In controlled trials published from 2000 to 2023

The search was updated on 17 March 2025 using the same strategy and keywords. The publication date was limited to articles published from 2023 to 2025.
Additional records downloaded: 2

8. **Ethos**
The search was conducted on 17 May 2023 and was updated on 17 March 2025.
Date searched: 17 May 2023
Records downloaded: 2

**Search strategy:**
Older adult* AND video AND exercise

The search was updated on 17 March 2025 using the same strategy and keywords. The publication date was limited to articles published from 2023 to 2025.
Additional records downloaded: 0

9. **ProQuest Thesis and Dissertation**
The search was conducted on 17 May 2023 and was updated on 17 March 2025.
Date searched: 17 May 2023
Records downloaded: 54

**Search strategy:**
abstract(Older adult* OR older person OR older people OR elder OR geriatric OR senior* OR aged 65) AND abstract(Exercise* OR training OR home OR physical activity OR program*) AND abstract(Video* OR taped OR DVD* OR website OR web-based OR remote OR tablet* OR ipad* OR smartphone* OR phone* OR youtube* OR computer* OR television* OR application* OR ehealth OR mhealth OR digital health) AND abstract(Balance OR strength OR fall* OR mobilit* OR physical perform* OR physical function* OR physical ability* OR functional perform* OR functional abilit*) AND abstract(random*)<br><br>Limiters: full text, dissertation & thesis, English language, published since 2000

The search was updated on 17 March 2025 using the same strategy and keywords. The publication date was limited to articles published from 2023 to 2025.
Additional records downloaded: 3
