# Supplementary material for: Effects of exercise programmes delivered using video technology on physical performance and falls in people aged 60 years and over living in the community: a systematic review and meta-analysis
Source: BMJ Open. 2025 Apr 30;15(4):e092775. doi: 10.1136/bmjopen-2024-092775 (PMC12049923; doi:10.1136/bmjopen-2024-092775)
Supplement: online supplemental file 2 [file bmjopen-15-4-s002.pdf]

Summary of the characteristics of included trials

Study author; country	Sample (n); % female; mean age	Exercise components	Duration; session length; frequency	Setting; exercise mode	Comparator	Video exercise; media; device	Physical performance measurements	Fall-related outcomes and measurements
Boongird et al (2017); Thailand	417; 86.6%; 74.08	Lower extremity strengthening, stretching, and balance training.	6 months; 60 min; 2-3x week	Home; individual; supervised	Non-exercise intervention	Entirely video; offline; Video Disk Recorder (VDR)	Strength (5-STS); Dynamic balance (TUG, BBS)	Fear of falling (Thai FES-I); Number of falls and fallers (self-recorded)
Caballer et al (2016); Spain	51; 69%; 69.1±4	Lower extremity strengthening, balance, mobility, flexibility, endurance.	4 months; 45 min; 3x week	Centre; group; supervised	No intervention	Entirely video; offline; DVD player	Mobility (TUG); Functional balance (BBS); Balance (OLS); Aerobic endurance (6MWT); Lower limb function (SPPB); Lower extremity strength (5-STS)	Not assessed
Chang et al (2023); Taiwan	167; 70.1%; 67.6±7.86	Resistance, static balance, dynamic balance, speed-walking.	4 months; 60 min; 2-3x week	Centre and home; both group and individual; supervised	Non-exercise intervention	Partially video (combined with face-to-face exercise); online; smartphone & LINE chat application	Upper limb strength (Grip strength); SPPB; static balance ability (OLS); physical agility (TUG); dynamic balance ability (functional reach)	Not assessed
Fyfe et al (2022); Australia	19; 67%; 69.8±3	Lower extremity strengthening, balance, functional tasks	1 month; 9 min; 3x day	Home; individual; unsupervised	No intervention	Entirely video; online; smartphone/tablet & website platform	Physical function (5-STS and 30s CST)	Not assessed
Haines et al (2009); Australia	50; 60.4%; 80.9±6.5	Muscle strength, balance	2 months; 13 min	Home; individual; supervised	No intervention	Entirely video; offline; DVD player	Balance (BOOMER); Strength (15s sit-to-stand); Mobility (2-minute walk test)	Fear of falling (ABC Scale); Number of falls (self-recorded)
Liang et al (2020); United Kingdom	30; 67%; 71.1±3.6	Functional tasks, muscle strength, balance, tai chi	1 month; 2x day	Home; individual; unsupervised	Non-exercise intervention	Entirely video; online; smartphone/tablet and website platform	Physical function (5-STS, 60s sit-to-stand, Leg standing balance)	Not assessed
Lytras et al (2022); Greece	150; 90.7%; 70	Lower extremity strengthening, balance, flexibility	6 months; 45 min; 5x week	Centre and home; both group and individual; supervised	Non-exercise intervention	Partially video (combined with face-to-face exercise); offline; TV or computer	Functional mobility (TUG); Static balance (4-stage balance); Leg strength (30s CST); Balance (BBS)	Fear of falling (short FES-I); Number of falls (self-recorded)

Continued

Study author; country	Sample (n); % female; mean age	Exercise components	Duration; session length; frequency	Setting; exercise mode	Comparator	Video exercise; media; device	Physical performance measurements	Fall-related outcomes and measurements
McAuley et al (2012); United States	260; 71.52%; 70.62±0.4	Muscle strength, balance, and flexibility.	6 months; 3x week	Home; individual; unsupervised	Non-exercise intervention	Entirely video; offline; DVD player	Functional performance (SPPB)	Not assessed
Meziere et al (2021); France	35; 83.3%; 90	Muscle strength, balance, functional tasks, joint mobilization exercises	3 months; 2x week	Home; individual; supervised	Non-exercise intervention	Partially video (combined with face-to-face exercise); offline; tablet	Walking and balance ability (TUG)	Absence of falls requiring medical care
Roberts et al (2017); United States	153; 73.6%; 70±4.98	Muscle strength, balance, and flexibility.	24 months follow up; 3x week	Home; individual; unsupervised	Non-exercise intervention	Entirely video; offline; DVD player	Functional performance (SPPB)	Not assessed
Vestergaard et al (2007); Denmark	53; 100%; 81±3.3	Muscle strength, balance, flexibility, and endurance	5 months; 26 min; 3x week	Home; individual; unsupervised	No intervention	Entirely video; offline; Video player	Functional ability (5-STS, 10-meter walking, standing balance test, PPT, Mob-T)	Not assessed
Wojcicki et al (2015); United States	237; 71.5%; 70.6±0.4	Muscle strength, balance, and flexibility.	12 months follow up; 3x week	Home; individual; unsupervised	Non-exercise intervention	Entirely video; offline; DVD player	Functional performance (SPPB)	Not assessed
Yamada et al (2011); Japan	84; 80.5%; 83±6.7	Muscle strength, balance, agility, and dual tasks.	6 months; 20 min; 2x week	Centre; group; supervised	No intervention	Entirely video; offline; DVD player	Functional fitness (TUG, 5-STS)	Not assessed
Suzuki et al (2024); Japan	15; 33.3%	Slow squats, one-legged stance	3 months; 15 min; daily	Home; individual; supervised	No intervention	Entirely video; online; smartphone & YouTube application	Muscle strength (Grip strength, knee extension strength); Balance capability (One-leg standing time with eyes open and with eyes closed)	Not assessed
Ferrari et al (2024); Italy	73; 49%; 66.89±5.9	Muscle strength and balance	6 months; 30 min; 3x week	Home; individual; supervised	No intervention	Entirely video; online; tablet & website platform	Balance (semi-tandem); Gait (10-meter walking)	Not assessed
Zhou et al (2025); China	116; 25%; 84.4±3.2	Muscle strength and balance	12 months; 30 min; 3x week	Home; individual; supervised	No intervention	Entirely video; online; smartphone & WeChat application	Grip strength; Usual gait speed; 5-STS; TUG	Rate of new falls: Fear of falling (FES-I)
