# Supplementary material for: Effects of exercise programmes delivered using video technology on physical performance and falls in people aged 60 years and over living in the community: a systematic review and meta-analysis
Source: BMJ Open. 2025 Apr 30;15(4):e092775. doi: 10.1136/bmjopen-2024-092775 (PMC12049923; doi:10.1136/bmjopen-2024-092775)
Supplement: Supplementary file 5 [file bmjopen-2024-092775.reviewer_comments.pdf]

# PEER REVIEW HISTORY

BMJ Open publishes all reviews undertaken for accepted manuscripts. Reviewers are asked to complete a checklist review form and are provided with free text boxes to elaborate on their assessment. These free text comments are reproduced below.

## ARTICLE DETAILS

### Title (Provisional)

Effects of exercise programmes delivered using video technology on physical performance and falls in people aged 60 years and over living in the community: A systematic review and meta-analysis

### Authors

Adliah, Fadhia; Hall, Abigail J.; Goodwin, Victoria; Lamb, Sarah

---

## VERSION 1 - REVIEW

---

| | |
|---|---|
| **Reviewer** | **1** |
| **Name** | **Arnold, Susanne** |
| **Affiliation** | **Warwick Medical School, Warwick Clinical Trials Unit** |
| **Date** | **03-Oct-2024** |
| **COI** | **I can confirm that I have previously published with one of the authors – Professor Sallie Lamb.** |

---

These are comments in relation to the systematic review and meta-analysis of the effects of exercise programmes delivered using video technology on physical performance and falls in people aged 60 years and over living in the community. i think this is a very important piece of work.

Abstract: a clear overview of the objectives, methods and results of the systematic review.

Introduction: a very clear introduction to the topic and rationale for the systematic review.

Methods:

PICO: I understand that people with specific diseases and conditions may present differently or have slightly different needs but as physical performance was your primary outcome can I just ask why exclude studies including these groups where exercise interventions may still have been targeting physical performance or function.

Results:

Page 6, line 48: Study characteristics - you report 14 articles were published between 2007 and 2023 and included but you have said you included 13 studies in the PRISMA and search outcome section.

Although it is clear from the text which direction the forest plots are favouring, this should be included on the bottom of each forest plot i.e. favours experimental or favours control.

Discussion:

Page 16, lines 5-12. I found this section of the paragraph slightly confusing.

I think these two sentences in particular need re-writing: "However, this increase was considerably smaller (0.96 seconds) than what would be expected based on the previous study's Minimal Clinically Important Difference (MCID)2 - I am not clear what previous study you mean.

"The MCID of the Timed-up-and-go (TUG) in older people that has ever been reported was 2.1 seconds." Do you mean this is the only paper that reports what an MCID for the TUG is?

I note that you discuss the benefits of using technology and video for providing exercise interventions (with which I agree) but do you think you need to mention anything about a potentially large group of older people who do not use technology and how this type of intervention excludes them. For example, someone aged 60 (the lower end of your inclusion age) may have very different technological experience to someone in their 90's - I acknowledge that as time goes on, all older people will be more technologically aware but I just include this as a point for consideration/thought.

---


| | |
|---|---|
| **Reviewer** | **2** |
| **Name** | **Zang, Wanli** |
| **Affiliation** | **Harbin Sport University** |
| **Date** | **05-Oct-2024** |
| **COI** | **None** |


---

This study effectively explores the use of video-delivered exercise programs to improve physical performance and prevent falls in community-dwelling older adults, providing valuable insights into this innovative intervention method. Its comprehensive meta-analysis and focus on vulnerable populations add to its strength, along with attention to adherence and satisfaction factors. However, the study has some limitations, such as the need for updated literature searches and a more structured discussion of key findings.

Abstract:

The objective should be concise and avoid unnecessary details. The word count for the abstract should be limited to 250 words.

Strengths and Limitations of this Study:

The points mentioned by the authors—"This systematic review was conducted following the Preferred Reporting Items for Systematic Reviews and Meta-Analyses (PRISMA) guidelines and followed a prespecified protocol registered on PROSPERO" and "The methodological quality of the included reviews was assessed using standardized measures"—are prerequisites for conducting an evidence-based study and should not be listed as strengths.

Similarly, the limitation "The trials are open-label, meaning that participants and therapists are aware of the intervention assignment" is a common issue in exercise intervention meta-analyses and should not be considered a limitation of this study. I suggest the authors modify the strengths and limitations section to focus on aspects specifically related to this study, such as first-time reported outcomes or unique insights.

Introduction:

The authors should review the logical flow of this section and ensure the arguments are clearly presented.

Methods:

The authors need to update the search strategy, as it has been over a year since the last search was conducted. Why was Web of Science not included in the search strategy?

While the inclusion criteria are described in detail, I would like the authors to double-check each included study to ensure all meet the population inclusion criteria. There may be studies that include mixed populations with participants who should be excluded, in which case these studies would need to be removed from the analysis.

Results:

This section requires significant revisions. The authors should restructure it according to the standard format used in mainstream meta-analyses. The flowchart should clearly indicate how many studies were included in the systematic review and how many in the meta-analysis. Figure 2 should also provide an explanation of each sub-figure, with labels such as Figure 2A and Figure 2B.

Discussion:

Consider adding subheadings in this section to improve clarity. It's important to succinctly summarize the study's key findings and deepen the discussion on those findings.

---

| | |
|---|---|
| **Reviewer** | **3** |
| **Name** | **Sánchez Romero, Eleuterio** |

**Affiliation** **Universidad Europea de Madrid Campus de Villaviciosa de Odón, Physiotherapy**

**Date** **14-Oct-2024**

**COI** **None**

---

The investigators have conducted a systematic review to synthesize evidence and evaluate the efficacy of exercise interventions delivered via instructional videos on physical performance in community-dwelling older adults aged 60 years and above. The primary objective was to assess improvements in muscle strength, balance, and mobility as key outcome measures.

The purpose of this review is to identify areas that require improvement or additional information to ensure the manuscript fully adheres to the systematic review reporting standards.

Title and Abstract

Title: The title identifies the study as a systematic review and meta-analysis, which aligns with PRISMA's recommendation.

Abstract: The abstract presents a structured format with objectives, methods, results, and conclusions. However, it would be enhanced by specifying the total number of studies included in the analysis and highlighting key outcomes (e.g., confidence intervals for the effect estimates). PRISMA recommends a more explicit outline of the search strategies and the method of risk of bias assessment, which is currently absent from the abstract.

Introduction

The rationale for the systematic review is described within the context of existing literature on exercise interventions for older adults. However, it would be advantageous to strengthen the justification regarding the necessity for a synthesis of evidence on video-based interventions specifically, particularly given the increasing significance of remote technologies post-COVID-19.

The objectives are clearly stated; however, it may enhance clarity to delineate the specific research questions being addressed (e.g., What is the impact of video-based interventions on fall prevention?) following the PICOS (Population, Intervention, Comparator, Outcome, Study design) framework.

Methods

The authors should update the search to the current date, as it is dated May 2023.

Eligibility criteria are well described, utilizing the PICO model to define inclusion/exclusion criteria (community-dwelling older adults aged ≥60, use of pre-recorded video exercise programs). However, a more comprehensive description of excluded studies would improve transparency (e.g., the rationale for excluding certain health conditions such as Parkinson's

disease). Information sources and search strategy: The databases and timeframe for the search are provided (MEDLINE, EMBASE, etc.); however, it would be advantageous to include the precise date of the final search and a supplementary table presenting the comprehensive search strategy. The search strategy should also encompass details of the grey literature searches, in accordance with PRISMA guidelines.

Study selection process is adequately described, with two reviewers independently screening titles and abstracts, resolving disagreements through a third reviewer. Inclusion of additional specifics regarding the potential utilization of automation tools during this process would enhance clarity.

Data collection process: The manuscript provides a satisfactory overview of the data extraction methodology. However, it would benefit from elucidating how missing data from studies were addressed. Additionally, PRISMA recommends including the form utilized for data extraction, which is currently not available.

Risk of bias assessment: The authors employed the ROB2 tool to assess the risk of bias, which is a commendable practice. However, further elucidation on the resolution of discrepancies between reviewers would enhance transparency. Providing an exemplar of the ROB2 results table for each included study might elucidate the levels of bias in the included studies.

Results

Study selection: The flow diagram is present, which is essential according to PRISMA. However, the study should provide more comprehensive details about the excluded studies, specifically elucidating why certain articles were excluded after full-text screening. PRISMA advises citing those excluded studies and providing rationales for their exclusion.

Study characteristics are reported in a table that summarizes relevant characteristics. Nevertheless, it could benefit from including a more comprehensive table that clearly delineates the interventions, comparators, and outcomes in a more accessible format for readers.

Risk of bias in studies: The manuscript discusses the overall bias of included studies, but presenting this data in a structured table format would enhance clarity, as PRISMA recommends. The bias assessment, although presented, could include a more detailed discussion about specific domains where high or low risks were identified and their potential impact on the study outcomes.

Results of individual studies: While the manuscript provides pooled effect sizes, it should also display summary statistics for each outcome per study. Additional visual representations, such as forest plots for all major outcomes, would comply with PRISMA's standards.

Synthesis of results: The synthesis of results is presented adequately, but more detail on how data was transformed or converted would improve clarity. For example, when utilizing

standardized mean differences, an explanation of the rationale for this approach and its application across studies would enhance transparency.

Certainty of evidence: The GRADE system is mentioned as being utilized, but a detailed table summarizing the certainty of evidence for each outcome is absent. Including this would align with PRISMA's recommendations for reporting certainty assessments. Discussion

The interpretation of results is generally well executed, with the authors discussing the primary findings in relation to previous research. However, the manuscript should explicitly state the limitations of the evidence provided, such as the limited number of included studies for certain outcomes, which affects the certainty of conclusions regarding falls and fear of falling.

Limitations of the review: The authors address some limitations of the review (e.g., the open-label design of most studies); however, a more comprehensive discussion of the limitations inherent to the review process (e.g., potential publication bias, language bias, reliance on self-reported data) would enhance the manuscript's rigor.

Implications for practice and research: The discussion on practical implications is adequate; however, expanding on the implications for future research, particularly the necessity for higher-quality randomized controlled trials, would enrich this section.

Recommended bibliography for Introduction and/or Discussion section

In the Introduction/Discussion section, it is recommended to incorporate and reference the following high-quality studies: DOI: 10.1097/TGR.0000000000000413 ; DOI: 10.3390/ijerph20054116 ; DOI: 10.3390/biology11071084

Your study proposes the examination of two papers: one analyzing the surgical treatment of the joint from an inflammatory perspective, and the other investigating osteoarthritis and sleep disturbances in relation to a joint other than the knee.:
DOI:10.1097/TGR.0000000000000337 ; DOI: 10.3390/biomedicines10092143

Conclusion

In conclusion, the systematic review by Fadhia Adliah et al. is comprehensive and methodologically sound. However, to fully adhere to the PRISMA guidelines, the authors should provide more detailed reporting in several key areas, including the search strategy, data extraction process, risk of bias assessments, and the certainty of evidence. Presenting these elements in a more structured, transparent manner will significantly enhance the clarity and completeness of the review. Furthermore, the manuscript would benefit from a more detailed discussion of limitations and implications for future research.

---

## VERSION 1 - AUTHOR RESPONSE

Reply to Reviewer 1
Dr. Susanne Arnold

We sincerely appreciate your insightful comments. Your comments have helped us refine and improve the overall quality of our work. Please allow us to respond to your comments.

Methods:

While we understand your suggestions to expand the population and involve people with specific disease, we feel that our approach has in line with our objectives. The primary goal of this review is to examine the effectiveness of video-based exercise on older adults compared to no intervention. People with specific conditions might have received other intervention to improve their functioning. Furthermore, this literature review is required to develop a video-based intervention for older people living in the community who do not have specific conditions. We believe that older persons with certain conditions (e.g Parkinson’s disease, stroke) might require more complex approach and advanced therapeutic exercise programmes.

Results:

Thank you for pointing this out. We included 13 studies in this review. A change has been made.

We agree with your suggestion to add caption regarding which direction the forest plots are favouring.

Discussion:

Thank you for bringing this to our attention. The study we cited on MCID is not the only one that reports on it. The sentence was little ambiguous - we have amended it.

We completely agree with your comment regarding exploring technological experience in this group of people and we were interested in investigating this before. However, the included studies did not give sufficient details about their participants’ perspective or attitudes about using technology for exercise, so we were unable to provide it throughout the discussion.

Reply to Reviewer 2

Dr. Wanli Zang

Thank you for your constructive and thoughtful comments. We appreciate your suggestions, which have allowed us to strengthen both the content and presentation of our manuscript.

Please allow us to address your comments.

Abstract:

The abstract has been revised accordingly.

Strength and limitations of this study:

We understand your concern regarding strength and limitations points. We discussed and decided that open-label should not be considered as a limitation in a review. Thus, we have updated this section.

After careful consideration, we respectfully disagree to remove PRISMA

guidelines from our strengths as we believe that PRISMA guidelines is a best practice for conducting and reporting systematic reviews and meta-analysis, and adhering to them enhances the transparency, rigor, and reproducibility of the research. However, we still found that some systematic reviews do not adhere to PRISMA guidelines. Thus, we feel that this can be count as a strong point in our review.

Methods:

In response to your comment regarding involvement Web of Science in the search strategy, we believe that the addition of Web of Science might be overlap with other databases, as it indexes a large proportion of articles covered by Pubmed and Embase. Furthermore, WoS covers a wide range of disciplines, whereas we sought greater specificity in health-related databases.

Results:

Thank you for pointing this out. We have made changes in the flowchart and adding sub-figure.

Discussion:

We appreciate your suggestions of adding subheadings in discussion. We feel that it is crucial to make discussion section not too long but enough to explain the key findings. Thus, we prefer a continuous narrative to maintain a more cohesive flow, rather than breaking it into subsections. We thought that a cohesive narrative without subheadings would provide a more fluid and continuous argument, making it easier for the reader to follow.

Reply to Reviewer 3

Dr. Eleuterio Sánchez Romero

We are thankful for detailed and constructive feedback you gave to our manuscript. Your input has incredibly improved the overall quality of our work. Allow us to respond to your comments.

Abstract:

Thank you for your suggestions. We have revised the abstract accordingly.

Introduction:

We appreciate your suggestions to elaborate further on certain aspects in the introduction. While we agree that additional details could be included, we believe that the current introduction adequately sets the stage for the study without overloading the reader with too much background. We prefer to keep the introduction succinct to maintain the flow of the manuscript.

While we understand the merit of using question format for research objectives, we intentionally presented the objectives in a narrative format to maintain a concise and direct description of the research aims. This format aligns with our intention to provide a clear overview of the study's

aims without posing them as specific questions.
Methods:
We understand your concern regarding the search date. The last search date we used remains valid as it covers a sufficient time frame to address the research question. We feel that the studies included in our review adequately represent the evidence up until the cutoff date. Furthermore, due to resource constraints and the timing of this submission, we were unable to conduct a full update of the systematic review search.
In response to your request of a supplementary table presenting the comprehensive search strategy, we have now provided more detailed information about search strategies in the supplementary material.
The rationale for excluding people with specific conditions (e.g. Parkinson’s disease) is because our primary goal of this review is to examine the effectiveness of video-based exercise on older adults compared to no intervention. People with specific conditions might have received other intervention to improve their functioning. Furthermore, this literature review is required to develop a video-based intervention for older people living in the community who do not have specific conditions. We believe that older persons with certain conditions (e.g Parkinson’s disease, stroke) might require more complex approach and advanced therapeutic exercise programmes.
Study selection:
The tools used during screening have been mentioned in the manuscript which are RAYYAN and EndNote.
Data collection:
Responding your concern about how missing data were addressed, we addressed missing data in quality assessment using Cochrane RoB2 tool to evaluate how missing data might affect the internal validity of individual studies. Studies with higher levels of missing data were rated as having a higher risk of bias.
Risk of bias assessment:
We understand your concerns about include the risk of bias assessment results table in the manuscript. While we agree that it could increase the transparency, we feel that presenting them in the main text would be too detailed at the same time and might be overwhelm the reader. Thus, we prefer to only provide a summary of risk of bias assessment in the manuscript, which we believe sufficiently addresses the concern without overwhelming the reader with excessive detail. We would be happy to provide the table upon request for interested readers.
Results:
We appreciate your suggestions for providing further details about the excluded studies as well as citing them. The full-text screening was performed on 103 studies, and the reasons for exclusion are presented in the PRISMA flow diagram. Citing 91 excluded articles is difficult due to the

limited length of a journal manuscript.
In regard to your comments about providing more details on the study's characteristics table, we agree that further information would enhance clarity. However, due to space constraints, we have limited the level of detail in this section. We feel that further elaboration would exceed the manuscript’s word limit without adding substantial value to the overall content. We believe that the current level of detail is appropriate for the scope of the manuscript, as it aligns with the focus of the research and is consistent with similar studies in the literature.
Risk of bias in studies:
Concerning your suggestions about adding result table for risk of bias in studies - We believe that Figure 2a, which demonstrates the risk of bias results for each study and its domains, is adequately representative and clear, thus adding another table would be redundant.
Results of individual studies:
Following your suggestion, we have added summary table for each outcome. Please see table 3.
Synthesis of results:
In response to your comment to provide an explanation for utilising MD or SMD - We have included this in the data analysis section. Data (continuous) gathered using the same measurement (for example, all mobility data is assessed using the TUG test) were computed using MD, while data collected using various measurements were calculated using SMD.
Certainty of evidence:
On page 13, we have Table 4 summarising the quality of evidence. We are unsure whether you mean this table or another type of table. Could you please help clarify your suggestion?
Discussion:
Thank you for your suggestions about stating the limitations of the evidence provided. We have made changes to the manuscript.
Limitations of the review:
We have revised the limitations of the review. Thank you for your suggestions.
Implications for practice and research:
We completely agree to add the necessity for higher quality randomised controlled trials.
Recommended bibliography:
This review is not related to surgical treatment of the joint and osteoarthritis. Could you please help clarify your suggestions?
Conclusion:
We sincerely appreciate your time and effort in reviewing our manuscript and providing such valuable feedback. We hope the revisions address your concerns, and we look forward to your further feedback.

**VERSION 2 - REVIEW**

**Reviewer** **1**
**Name** **Arnold, Susanne**
**Affiliation** **Warwick Medical School, Warwick Clinical Trials Unit**
**Date** **03-Feb-2025**
**COI**

Thank you so much for responding to my comments and suggestions. I will recommend that your paper is now accepted for publication.

**Reviewer** **2**
**Name** **Zang, Wanli**
**Affiliation** **Harbin Sport University**
**Date** **04-Mar-2025**
**COI**

The author has revised the manuscript based on the suggested modifications and provided a response. I have no additional comments for further revision.

**Reviewer** **3**
**Name** **Sánchez Romero, Eleuterio**
**Affiliation** **Universidad Europea de Madrid Campus de Villaviciosa de Odón, Physiotherapy**
**Date** **18-Feb-2025**
**COI**

I appreciate the opportunity to review your systematic review manuscript. I commend the effort put into synthesizing the available literature on this important topic. The study follows a structured approach and provides valuable insights. However, after a detailed assessment based on PRISMA 2020 guidelines, I have identified areas that require improvement to enhance clarity, methodological transparency, and reproducibility. Below is a detailed evaluation of your manuscript with constructive feedback.

Reviewer Report

Title & Abstract:

The title is appropriate but could be refined for clarity.

The abstract needs minor adjustments to align with PRISMA reporting standards.

Introduction:

The background is well-developed, but further justification of research questions is needed.

Methods:

The eligibility criteria are clearly outlined.

Search strategy details are insufficient.

Statistical methods require more elaboration.

Risk of bias assessment should be expanded.

Results:

Figures and tables are informative but need further annotations.

Subgroup analyses should be reported in greater depth.

Discussion:

Limitations and future research directions should be elaborated.

The relevance of findings to clinical practice should be highlighted more explicitly.

Conclusion:

Well-stated but could be strengthened with practical implications.

Recommended Bibliography Section

To further support the discussion and findings of this manuscript, we suggest including the following references. These studies contribute relevant insights into frailty, neuromuscular rehabilitation, and aging-related physiological challenges.

Telemedicine Interventions for Postural Instability in Parkinson's Disease

DOI: 10.1097/TGR.0000000000000413

Justification: Given the increasing use of digital health tools in aging populations, this review highlights the role of telemedicine in managing balance and neuromuscular function, which are key factors in frailty prevention and rehabilitation.

DOI: 10.3390/biology11071084

Justification: This retrospective cohort study examines the effects of multicomponent exercise programs on post-COVID-19 older adults recovering from ICU care. It offers strong evidence supporting exercise-based interventions for frailty management.

These studies will enrich the manuscript by providing robust, evidence-based context related to frailty and rehabilitation strategies, aligning with the current research topic.

Frailty Detection in Aging Populations

DOI: 10.18632/aging.206162

Justification: This multicenter big-data-based study protocol focuses on early detection and classification of frailty, aligning with the manuscript's emphasis on identifying risk factors and preventative strategies for age-related musculoskeletal decline.

Frailty Detection and Big Data

DOI: 10.21203/rs.3.rs-4190311/v1

This multicenter cohort study protocol outlines the use of big data analytics to refine frailty detection, aligning with the present study's aims of integrating advanced methodologies for patient assessment.

Final Recommendation

✅ Major revisions required before acceptance.

🔷 The study is valuable, but methodological reporting and discussion of biases need improvement.

I appreciate the authors' work on this topic and look forward to seeing an improved version of the manuscript that fully aligns with PRISMA 2020 guidelines.

---

## VERSION 2 - AUTHOR RESPONSE

Reply to Reviewer 3's comments

Thank you for your thoughtful and constructive feedback. We appreciate the time and effort you have dedicated to reviewing our manuscript. Please allow us to address all the comments.

Reviewer report

Title & Abstract: The title is appropriate but could be refined for clarity.

The abstract needs minor adjustments to align with PRISMA reporting standards.

Response: The title effectively represents key elements, including population, intervention, outcomes, and study design, ensuring clarity. The abstract has been refined to better align with PRISMA reporting standards.

Introduction: The background is well-developed, but further justification of research questions is needed.

Response: The introduction has been refined to ensure a logical flow, transitioning smoothly from the general benefits of exercise to the specific focus on video-based interventions. It clearly establishes the problem, presents video-based interventions as a potential solution, and provides a well-justified rationale and research question for this systematic review.

Methods: 1) Search strategy details are insufficient. 2) Statistical methods require more elaboration. 3) Risk of bias assessment should be expanded.

Response: A full search strategy has been provided in the supplementary file. All statistical methods used have already been presented. The risk of bias assessment has been thoroughly explained in the manuscript under the methodological quality assessment section.

Results: 1) Figure and tables are informative but need further annotations. 2) subgroup analyses should be reported in greater depth.

Response: Figure legends are provided at the end of the manuscript. The tables are already clear and do not require further annotations. Subgroup analysis could not be performed due to the limited number of studies, and this limitation has been acknowledged in the study limitation section.

Discussion: 1) Limitations and future research directions should be elaborated. 2) The relevance of findings to clinical practice should be highlighted more explicitly.

Response: The discussion has been revised to provide a more comprehensive overview, incorporating limitations, future research directions, and explicit implications for clinical practice.

Conclusion: Well-stated but could be strengthened with practical implications.

Response: Practical implications have been added to strengthen the conclusion.

Recommended Bibliography Section: To further support the discussion and findings of this manuscript, we suggest including the following references. These studies contribute relevant insights into frailty, neuromuscular rehabilitation, and aging-related physiological challenges.

Response: We appreciate your recommendations. After careful review, we can cite Telemedicine Interventions for Postural Instability in Parkinson’s Disease, as it aligns with the scope of our study. However, the other suggested references focus on broader topics such as frailty detection and post-COVID rehabilitation, which fall outside the primary focus of our manuscript.
